# The Impact of Morbidity and Disability on Attendance at Organized Breast Cancer–Screening Programs: A Systematic Review and Meta-Analysis

**DOI:** 10.1158/1055-9965.EPI-21-1386

**Published:** 2022-05-05

**Authors:** Lorna McWilliams, Samantha Groves, Sacha J. Howell, David P. French

**Affiliations:** 1 Division of Psychology and Mental Health, School of Health Sciences, Manchester Center for Health Psychology, Faculty of Biology, Medicine and Health, University of Manchester, Manchester, United Kingdom.; 2 NIHR Manchester Biomedical Research Center, Manchester Academic Health Science Center, Manchester University NHS Foundation Trust, Manchester, England.; 3 Division of Cancer Sciences, School of Medical Sciences, Faculty of Biology, Medicine and Health, University of Manchester, Manchester, United Kingdom.

## Abstract

Individuals with morbidity experience worse breast cancer outcomes compared with those without. This meta-analysis assessed the impact of morbidity on breast cancer–screening attendance and subsequent early detection (PROSPERO pre-registration CRD42020204918). MEDLINE, PsychInfo, and CINAHL were searched. Included articles published from 1988 measured organized breast-screening mammography attendance using medical records by women with morbidity compared with those without. Morbidities were assigned to nine diagnostic clusters. Data were pooled using random-effects inverse meta-analyses to produce odds ratios (OR) for attendance. 25 study samples (28 articles) were included. Data were available from 17,755,075 individuals, including at least 1,408,246 participants with one or more conditions;16,250,556 had none. Individuals with any morbidity had lower odds of attending breast screening compared with controls [*k* = 25; OR, 0.76; 95% confidence interval (CI), 0.70–0.81; *P* = <0.001; *I*^2^ = 99%]. Six morbidity clusters had lower odds of attendance. The lowest were for neurological, psychiatric, and disability conditions; ORs ranged from 0.45 to 0.59 compared with those without. Morbidity presents a clear barrier for breast-screening attendance, exacerbating health inequalities and, includes a larger number of conditions than previously identified. Consensus is required to determine a standardized approach on how best to identify those with morbidity and determine solutions for overcoming barriers to screening participation based on specific morbidity profiles.

## Introduction

With increasing incidence rates (1), breast cancer is the most common cancer affecting females globally (2). Women with breast cancer and who have pre-existing conditions have worse outcomes (3). This may include physical conditions, as well as disabilities, although co-occurrence is generally used when describing individuals with the latter conditions or multiple conditions, referred to as multimorbidity. As one might expect, all-cause mortality is increased in individuals with breast cancer and a range of morbidities compared with those with breast cancer alone (4). For example, overall survival was 28% less at 5-years for individuals with breast cancer and dementia compared with those with breast cancer and no comorbidity (4). However, the presence of high morbidity burden, as measured by the impact of morbidity on non–cancer-related mortality, results in nearly four times greater odds of being diagnosed with distant metastases when compared with those with no morbidities (5, 6).

One route by which morbidity may contribute to late breast cancer detection is the influence on attendance at mammographic screening. Organized, population-based breast cancer–screening programs reduce breast cancer–related mortality by approximately 20% (7). However, a systematic review of breast screening in women over 65 years found that attendance for mammography in the United States decreased with increasing multimorbidity (8), assessed by Charlson Comorbidity Index (CCI) scores (ref. 9; which weights the relative impact of different conditions and levels of severity to predict the relative risk of one-year mortality). By contrast, the same review found that the greater absolute number of health conditions a person had was weakly associated with increased odds of attendance [odds ratio (OR), 1.17; 95% confidence intervals (CI), 1.00–1.36]. A second review of breast-screening participation, including results from only three studies, reported that having any morbidity was associated with reduced likelihood (OR, 0.66; 95% CI, 0.44–0.48) of attending breast screening (10). Both systematic reviews included predominantly US-based studies where only opportunistic screening is provided. Therefore, factors other than morbidity, such as financial barriers to screening, may have a greater influence on attendance (11). Combining studies of both opportunistic and organized screening programs is problematic in that morbidity may lead to screening attendance due to increased healthcare contact in areas where opportunistic screening is used. Many composite comorbidity scores such as CCI, as used in both reviews, do not include conditions linked with reduced screening attendance such as psychiatric conditions or disabilities (12, 13). In addition, composite scores do not allow for individual morbidity types to be studied, which is important to identify where interventions should be developed for individuals with those conditions. Finally, both reviews included studies using self-reported screening attendance that is often overestimated (14).

Several other systematic reviews of breast-screening attendance have focused on the impact of single conditions, demonstrating that compared with those without morbidity, attendance is less likely for individuals with mental illness (OR = 0.65; ref. 12), dementia or cognitive impairment (relative risk = 0.81; refs. 15, 16) and, diabetes (OR = 0.81; ref. 17). Although these reviews importantly quantify the impact of different conditions, they do not allow for comparisons between morbidity or disability types. A recent overview grouped individual conditions together where possible (18). However, full systematic methods were not shown and quantitative analyses were not conducted. Thus, between-condition comparisons or adjusted analyses based on study quality were not presented. Given the various weaknesses of all previous reviews in this area, this systematic review aimed to investigate the relative impact of individual morbidities and multimorbidity on breast cancer screening and thus, the potential for breast cancer early detection.

## Materials and Methods

### Search strategy and selection criteria

This review was pre-registered with PROSPERO, CRD42020204918 and followed the Preferred Reporting Items for Systematic Reviews and Meta-analyses (PRISMA) 2020 guidelines (19).

Included studies were research articles published from 1988 onwards when organized breast cancer–screening programs were introduced (20). Included articles assessed breast cancer–screening attendance in adult participants (over 18 years) systematically invited to organized population-based breast cancer–screening programs with (i) either morbidity or multimorbidity and, (ii) without any such specified conditions. Studies were eligible if morbidity was assessed using either a single condition or a validated composite score, such as CCI. The following studies were excluded when: (i) morbidity was diagnosed after cancer diagnosis; (ii) screening attendance was self-reported; (iii) attendance at opportunistic screening was examined; and (iv) crude data or ORs (adjusted or unadjusted) were not provided (authors were not contacted).

An electronic database search using MEDLINE, PsycInfo, and CINAHL was completed in September 2020 using tailored key words and subject headings (see Supplementary File for full search terms). Forwards and backwards citation, relevant review and author reference list searches identified additional articles. After deduplication, titles and abstracts were reviewed for exclusion by one author (S. Groves); a second author (L. McWilliams) independently reviewed 20% of these, yielding 97% agreement. Full-text assessment was conducted by both authors, with 95% agreement. Conflicts were resolved through discussion and where necessary with the other study authors.

### Data analysis

For each study, relevant information was extracted into an Excel document. Where necessary, crude data were used to calculate unadjusted ORs, for example, in studies that reported incidence rate or prevalence proportion ratios (21). When ORs were presented for non-attendance, the inverse was calculated. In studies with overlapping samples, or if multiple individual morbidities compared with the same or potentially overlapping control groups were presented, published data were used to compute a single summary score with a control group consisting of individuals without morbidity. Where data were presented as full, partial, and non-attendance, full attendance versus nonattendance data were extracted. Data were extracted by one author (S. Groves) and verified by (L. McWilliams).

Study quality was assessed using the widely used and adaptable Newcastle-Ottawa Scale (NOS; ref. 22) where a maximum of 9 points can be assigned to studies of highest quality. One author assessed study quality (S. Groves) in discussion with a second author (L. McWilliams). Articles with scores of seven or more were deemed to be high quality (see Supplementary Table S1 for quality assessment scores of included studies). Publication bias was assessed using Meta-Essentials (23) to produce a funnel plot, including studies that provided raw data and using the Begg–Mazumdar Kendall's tau and Egger's tests (24).

Analyses were conducted using Review Manager 5.3. Inverse-variance meta-analyses using a random effects model were used to pool ORs across studies, as heterogeneity surrounding study design and individual conditions studied were expected (25). The main outcome was breast cancer–screening attendance and the main analysis examined differences in screening rates between individuals with morbidity and controls. Additional analyses examined the association of a composite score of morbidity and multimorbidity on breast cancer–screening attendance. Heterogeneity was assessed using the *I*^2^ statistic, where a value of greater than 50% was considered indicative of substantive heterogeneity (26).

Predefined sensitivity analyses were conducted to examine the effect of unadjusted ORs, self-reported morbidities, and lower quality scores (NOS score of <7) on the overall outcome. Planned moderator analyses examined the impact of individual morbidity diagnostic clusters, geographical clusters, and participant type (research participants or general population). Morbidity clusters were based on the ACE-27 measure, commonly used to record pre-existing conditions in newly diagnosed patients with cancer: Cardiovascular; respiratory; gastrointestinal; renal endocrine; neurological; psychiatric and malignancy (27). The rheumatologic and immunological studies were collapsed on the basis of the limited number of relevant studies in the latter cluster as were renal and endocrine studies. Obesity was included within the cardiovascular cluster and anxiety/affective disorders included in psychiatric, along with substance misuse. Disability, including physical, intellectual and developmental conditions, was added as a ninth cluster. Although not formally recorded in the ACE-27, disabilities are generally included in medical notes (e.g., visual impairment, deafness) and may affect screening attendance (28). Exploratory analyses were conducted to investigate unexplained heterogeneity.

### Data availability

The data extracted and used in analysis are available in Supplementary Table S2.

## Results

**Figure 1.**
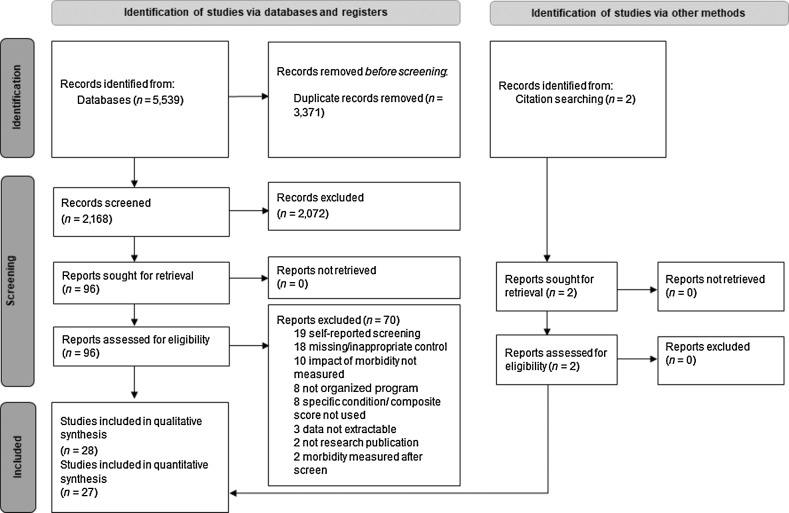
Study selection PRISMA diagram.

Of 2,168 results identified from the database searches, 28 articles were eligible for qualitative synthesis, and 25 samples (from 27 articles) were eligible for quantitative synthesis (see Fig. 1; Supplementary Table S3 lists reasons for article exclusion; Supplementary File for included and excluded references).

Characteristics of included studies are shown in Table 1, with further characteristics displayed in Supplementary Table S2. Of the included 28 studies, 14 examined the impact of one condition whereas 14 included multiple conditions. All nine morbidity clusters were represented in analyses.

**Table 1.** Frequency of key characteristics of included studies.

Characteristic	Number of studies
*Geographical location*	
Canada	9
United Kingdom	7
Scandinavia	8
Other	3
**One versus multiple conditions studied**	**Number of studies**
One	14
Multiple	14
**Morbidity composite score assessed**	**Number of studies**
Yes	4
**Morbidity cluster**	**Number of studies**
Neurological	6
Psychiatric	9
Disability	7
Renal and endocrine	6
Respiratory	5
Gastrointestinal	5
Malignancy	7
Rheumatologic and immunological	5
Cardiovascular	8
**Multimorbidity assessed**	**Number of studies**
Yes	5 (3 suitable for meta-analysis)
**Morbidity measurement**	**Number of studies**
Self-reported	4
Medical records/admin database	21
Measured by researchers	2
**Number of screening opportunities**	**Number of studies**
One	21
Multiple (6–23 years)	6
**Adjusted values used in main analyses**	**Number of studies**
Yes	9
**NOS quality score**	**Number of studies**
5–6	6
7–9	21

**Figure 2.**
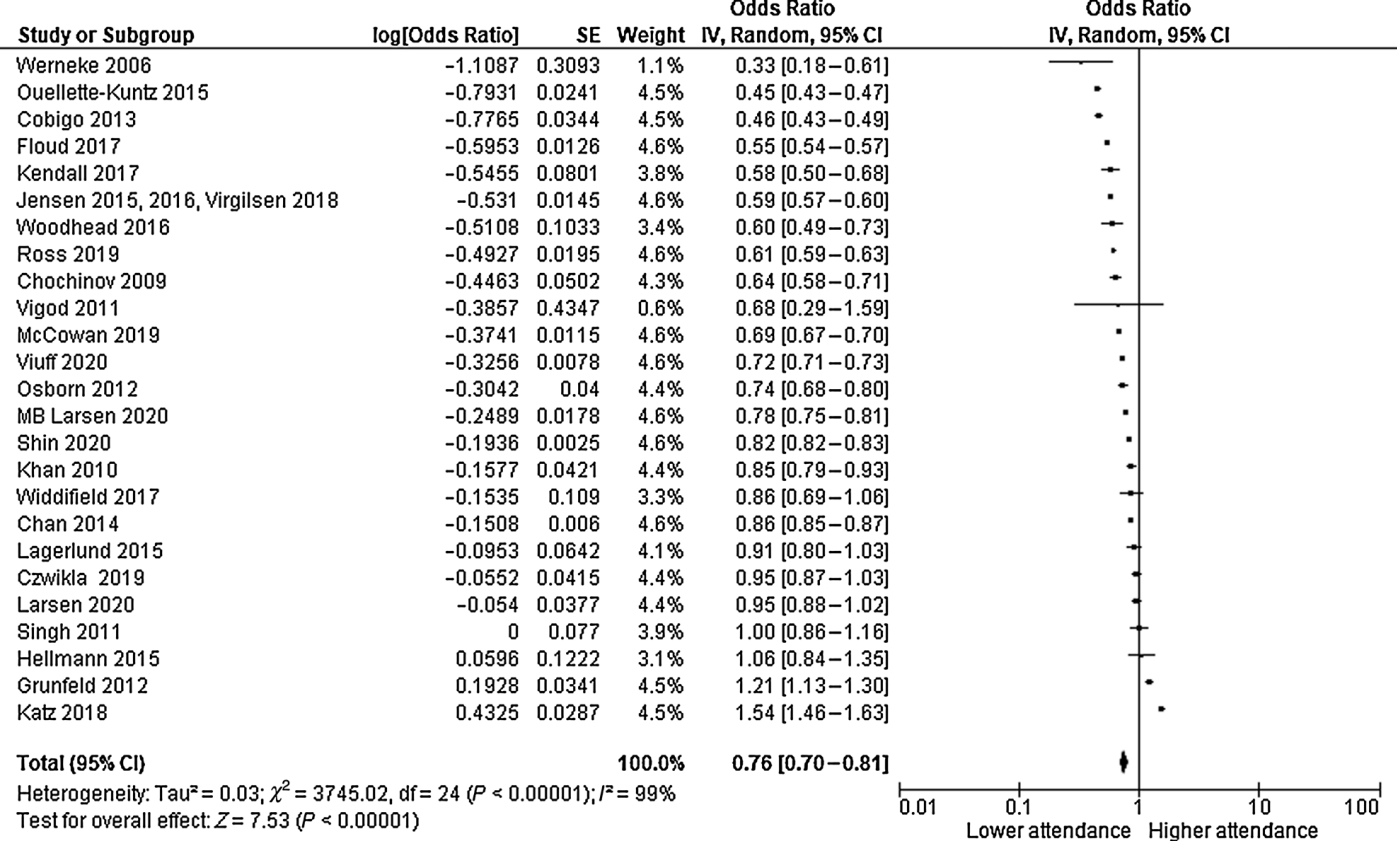
Forest plot estimating impact of morbidity on mammographic breast cancer–screening attendance.

Overall, individuals with morbidity had significantly lower odds of attending breast cancer screening than those without (Fig. 2), *k* = 25; OR, 0.76; 95% CI, 0.70–0.81; *P* ≤ 0.001; *I*^2^, 99%.

**Figure 3.**
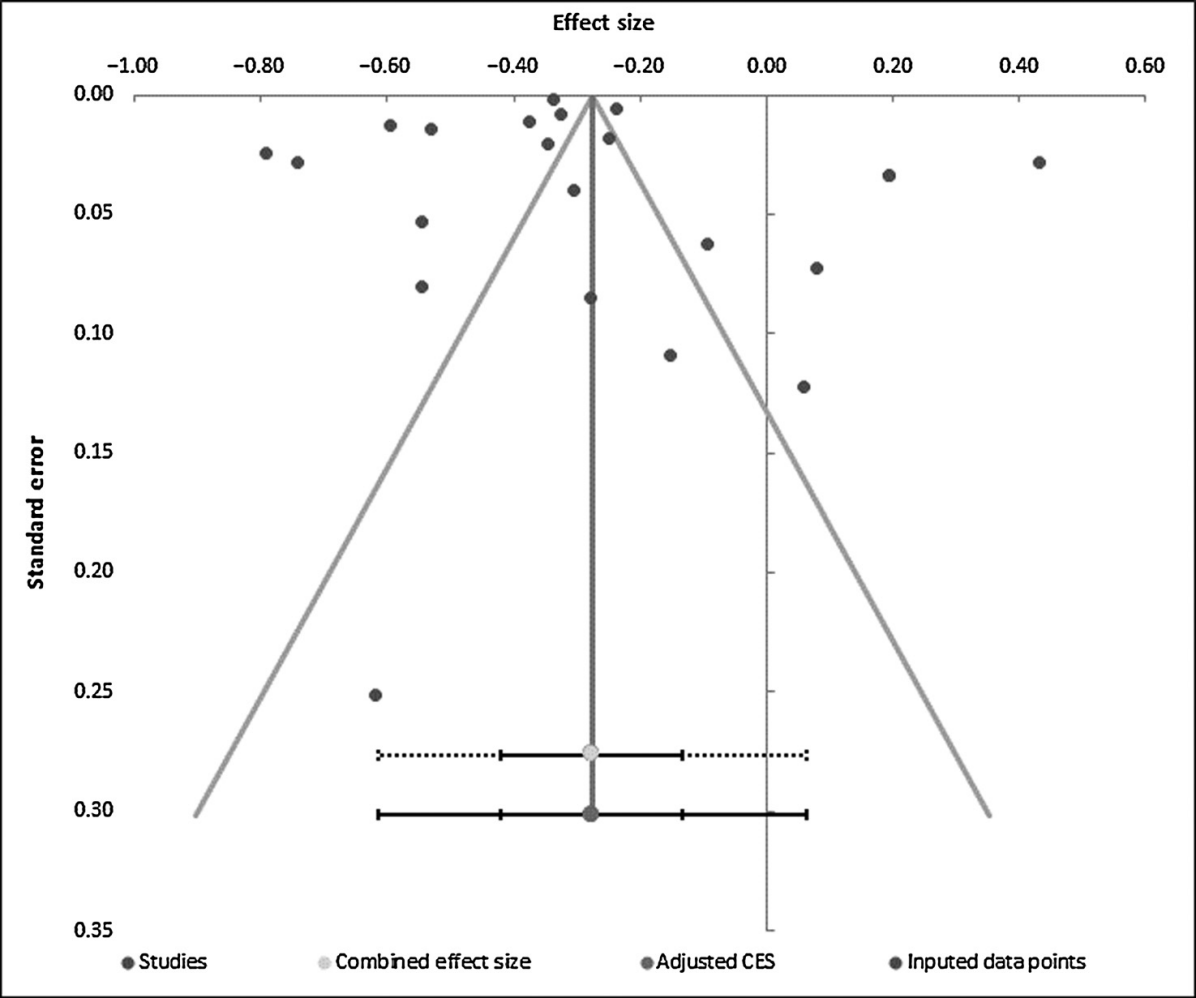
Funnel plot of standard error by odds ratio using raw data available from 22 studies.

Results remained virtually unchanged when omitting studies where morbidity was self-reported (*k* = 21; OR, 0.77; 95% CI, 0.72–0.83; *P* ≤ 0.001; *I*^2^, 99%), articles with <7 NOS score (*k* = 19; OR, 0.73; 95% CI, 0.67–0.79; *P* ≤ 0.001; *I*^2^, 99%), and unadjusted results (*k* = 9; OR, 0.76; 95% CI, 0.71–0.81; *P* ≤ 0.001; *I*^2^ 98%). In addition, results were similar after removing studies assessing attendance at multiple screening opportunities (*k* = 19; OR, 0.69; 95% CI, 0.64–0.75; *P* ≤ 0.001; *I*^2^, 99%) and those that included multiple conditions (*k* = 14; OR, 0.72; 95% CI, 0.62–0.84; *P* ≤ 0.001; *I*^2^, 99%). A funnel plot (Fig. 3), Egger's test (*P* = 0.942) and Kendall's *t* = 0.09 (*P* = 0.573) revealed no publication bias for the primary outcome in 22 studies (five studies did not provide raw data required for assessing publication bias). A trim and fill analysis was therefore not conducted.

Of four studies reporting the impact of CCI scores (characteristics and results summarized in Table 2), three were conducted in Denmark (29–31), and one in the UK (32). Two studies reported results as CCI 1 versus ≥2 (29, 30), and the remaining used 1–2 versus ≥3 (31, 32) compared with no morbidity as comparison. Thus, meta-analyses were not appropriate for this outcome. All studies reported that with increasing morbidity score, the odds of screening attendance decreased. Three studies reported that any Charlson score resulted in reduced attendance compared with those without (29–31), whereas one reported that individuals with a score of 1–2 were more likely to attend than those with either a score of 0 or 3 or more (32).

**Table 2.** Characteristics of studies reporting impact of Charlson Comorbidity Index (CCI) score on attendance.

Study	Country	Study design	CCI Score	Odds ratio (95% CIs)
Larsen 2018	Denmark	Cohort	1	OR, 0.74 (0.70–0.78)
			≥2	OR, 0.54 (0.51–0.58)
McCowan 2019	UK	Cohort	1–2	AOR, 1.05 (1.02–1.08)
			≥3	AOR, 0.75 (0.72–0.78)
Virgelsen 2018	Denmark	Cohort	1	OR, 0.82 (0.79–0.85)
			≥2	OR, 0.57 (0.55–0.60)
Viuff 2020	Denmark	Cross-sectional	1–2	OR, 0.79 (0.78–0.80)
			≥3	OR, 0.42 (0.41–0.44)

Abbreviations: AOR, adjusted odds ratio; CI, confidence intervals.

**Figure 4.**
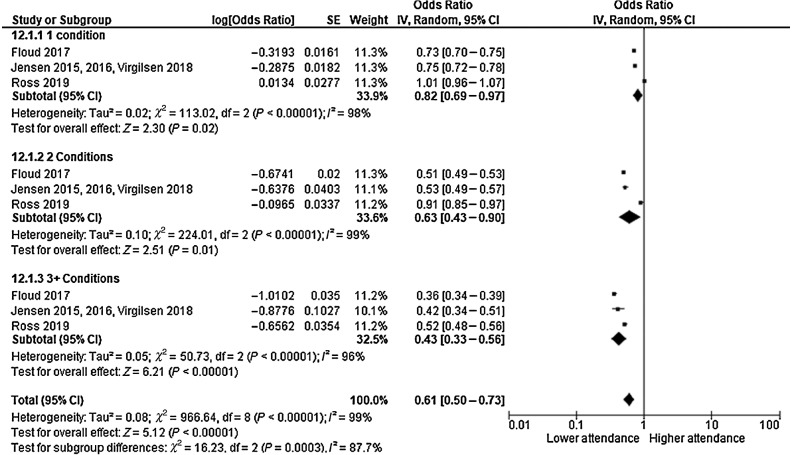
Forest plot estimating impact of multimorbidity on mammographic breast cancer–screening attendance.

Meta-analyses examined multimorbidity and breast cancer–screening attendance with three cohort studies, two conducted in the UK (13, 33) and one in Denmark (34). Two studies (13, 34) examined the impact of having 1, 2, or 3 or more conditions, whereas the other (33) presented results as 1, 2, 3 or, 4 or more. Data were merged for those with at least 3 conditions using crude data. Any count of morbidities was associated with reduced screening attendance (*k* = 3; OR, 0.61; 95% CI, 0.50–0.73; *P* ≤ 0.001; *I*^2^, 99%) with significant subgroup differences; a greater number of conditions was associated with lower attendance (Fig. 4).

Subgroup analyses showed that individual morbidity clusters significantly modified the overall effect (see Table 3). Individuals with neurological conditions had the lowest odds of attending breast cancer screening (*k* = 6; OR, 0.45; 95% CI, 0.31–0.65; *P* ≤ 0.001; *I*^2^, 99%) followed by those with psychiatric conditions (*k* = 9; OR, 0.57; 95% CI, 0.48–0.68; *P* ≤ 0.001; *I*^2^, 99%), disabilities (*k* = 7; OR, 0.59; 95% CI, 0.47–0.74; *P* ≤ 0.001; *I*^2^ 100%), renal and endocrine conditions (*k* = 6; OR, 0.63; 95% CI, 0.41–0.97; *P* = 0.04; *I*^2^ 100%), respiratory conditions (*k* = 5; OR, 0.72; 95% CI, 0.53–0.98), *P* = 0.04; *I*^2^, 100%), and malignancy (*k* = 7; OR, 0.81; 95% CI, 0.71–0.91; *P* ≤ 0.001; *I*^2^ 98%), respectively. The lower rates for gastrointestinal (*k* = 5; OR, 0.75; 95% CI, 0.52–1.08; *P* = 0.12; *I*^2^, 99%), cardiovascular (*k* = 8; OR, 0.94; 95% CI, 0.77–1.16; *P* = 0.58; *I*^2^, 99%), and rheumatologic and immunological conditions (*k* = 5; OR, 0.89; 95% CI, 0.69–1.15; *P* = 0.39; *I*^2^, 98%) were not statistically significant. No cluster was associated with increased odds of attendance. Considerable heterogeneity remained in all morbidity clusters.

**Table 3.** Subgroup random effect meta-analyses of morbidity cluster, study location, and participant type.

	Number of studies (Samples)	Odds ratio (95% CIs)	*P*	*I*²	*P* value of subgroup differences
**Morbidity cluster**					
Neurological	6	0.45 (0.31–0.65)	<0.001	99%	<0.001
Psychiatric	9	0.57 (0.48–0.68)	<0.001	99%	
Disability	7	0.59 (0.47–0.74)	<0.001	100%	
Renal and endocrine	6	0.63 (0.41–0.97)	0.04	100%	
Respiratory	5	0.72 (0.53–0.98)	0.04	100%	
Gastrointestinal	5	0.75 (0.52–1.08)	0.12	99%	
Malignancy	7	0.81 (0.71–0.91)	<0.001	98%	
Rheumatologic and immunologic	5	0.89 (0.69–1.15)	0.39	98%	
Cardiovascular	8	0.94 (0.77–1.16)	0.58	99%	
**Geographical area of study**					
Canada	9	0.71 (0.55–0.92)	0.01	99%	0.03
UK	7	0.65 (0.58–0.73)	<0.001	98%	
Scandinavia	8 (6)	0.80 (0.71–0.90)	<0.001	98%	
Other	3	1.06 (0.70–1.61)	0.77L	100%	
**Participant type**					
Research participants	6	0.77 (0.64–0.93)	0.007	98%	0.82
Non-research participants	21(19)	0.75 (0.70–0.81)	<0.001	99%	

Abbreviation: CI, Confidence Intervals.

Exploratory analyses omitting self-reported morbidity, unadjusted ORs, lower quality studies, and studies, including multiple screening opportunities, had minimal effect on within cluster heterogeneity. Analyses omitting obesity and hypertension from the cardiovascular cluster (*k* = 5; OR, 0.86; 95% CI, 0.71–1.03; *P* < 0.001; *I*^2^, 99%), substance misuse from psychiatric (*k* = 9; OR, 0.57; 95% CI, 0.49–0.67; *P* < 0.001; *I*^2^, 99%) and examining individual conditions alone (e.g., diabetes), also had no effect given the few studies available. Given the variability in disability type across studies (see Supplementary Table S1), an analysis stratified by type (intellectual and developmental, mobility, speech and language, and sensory) revealed no significant between-group differences (*P* = 0.16).

## Discussion

This systematic review found that comorbidity is associated with reduced odds of attendance, with the greatest odds of non-attendance for individuals with neurological (OR = 0.45) or psychiatric conditions (OR = 0.57) and disabilities (OR = 0.59), respectively. Furthermore, odds of attendance reduced with increasing multimorbidity, particularly when individuals have three or more conditions. Substantial heterogeneity was found across all findings, unexplained by study quality, adjustment for covariates, morbidity measurement type and clustering of morbidity.

Some differences between morbidity cluster and screening attendance may be explained by the cognitive and functional impact of specific morbidities. For example, certain neurological conditions and severe disabilities are likely to affect activities of daily living, which require caregiver support, including both the decision to participate and the practicalities of attending screening appointments compared with cardiovascular conditions such as hypertension. A previous review showed that dementia and cognitive impairment are associated with lower odds of cancer-screening attendance (15). The present review further demonstrates the impact of other neurological conditions, indicating a much greater likelihood of non-attendance (OR = 0.45 compared with OR = 0.81; ref. 15), which have not been considered previously. Reasons for reduced attendance among individuals with neurological conditions and disabilities may include individual-level circumstances such as low perceived importance by caregivers (35, 36), limited knowledge of breast cancer, and screening procedures (37, 38). In addition, systems-level factors such as inflexibility of diagnostic equipment and facilities (39) and a greater likelihood of individuals with these conditions residing in care homes, introduces further barriers to screening access and could reflect competing healthcare priorities.

Consistent with previous findings that those with psychiatric conditions have reduced odds of screening attendance (12, 40, 41), we have extended this by demonstrating that these disparities persist when considering organized screening programs. ORs of non-attendance in the present review were greater (OR = 0.57) than two previous meta-analyses that considered mental illness in organized and opportunistic screening programs [OR = 0.65 (ref. 12) and OR = 0.71 (40)]. A third meta-analysis found similar, but greater reduced odds (OR = 0.50) of attendance for women with schizophrenia (41). The notable impact of schizophrenia on reduced attendance is likely related to the severity of this particular psychiatric condition. Furthermore, the strength of association for odds of non-attendance for psychiatric morbidity is greater than for many physical condition clusters included in this review. Low value in one's own health, fear of leaving home, and another systems-level disadvantage related to lack of screening invitation during inpatient care have been demonstrated as key barriers by individuals with mental health difficulties (42).

The finding that the odds of individuals with current or previous malignancy attending screening lower than those with no morbidity (OR = 0.81) conflicts with previous reviews focusing on cancer survivors (43, 44). However, previous reviews primarily included US-based studies (where breast screening is generally discussed in primary care) and did not include individuals with a current cancer diagnosis that has been demonstrated to be negatively associated with screening attendance (45, 31). Lower odds of attendance (OR = 0.63) among those with renal and endocrine conditions adds to a previous meta-analysis demonstrating the association of diabetes and decreased attendance (17). As this review included conditions other than diabetes, this may explain the greater odds of non-attendance for this cluster compared with focusing on diabetes alone (OR = 0.83; ref. 17). Similarly, the OR of attendance was lower (OR = 0.72) than those with no morbidity for the respiratory cluster. It may be that the studies included in the respiratory cluster included individuals with more severe conditions, that is, chronic obstructive pulmonary disease with health demands that compete with travelling to attend breast screening. However, these studies did not assess severity of conditions.

There was no significant association between cardiovascular, gastrointestinal or rheumatologic/immunologic comorbidity clusters and odds of screening attendance. This did not appear to be due to within-cluster variability of types of conditions; exploratory analyses removing conditions did not affect results. However, the statistical power of analyses should be considered in relation to numbers of participants with each specific condition included within a cluster (e.g., only one study in the cardiovascular cluster measured the impact of cardiac arrhythmias). In addition to individual comorbidity clusters, this review demonstrated that multimorbidity is associated with reduced odds (OR = 0.61) of attendance compared with those without morbidity, which reduces to OR = 0.43 for those with three or more conditions. This contrasts with a previous review that found only a weak positive association (OR, 1.17; 95% CI, 1.00–1.36; ref. 8); however, participants self-reported screening attendance and the American healthcare system are unique in how it offers screening.

High levels of heterogeneity were found in all analyses, which were largely unchanged after conducting pre-planned sensitivity and additional exploratory analyses. Although adjusted ORs were used where possible for the primary outcome, to account for variance due to age; for example, it was not possible to use these in the morbidity cluster analyses. Exploratory analyses included an attempt to refine morbidity clusters further; however, it was not possible to conduct analyses based on specific conditions in some clusters due to the small number of eligible studies. It is likely that differences in identifying and defining levels of morbidity or multimorbidity, as well as suspected high levels of variability within individual multimorbidity profiles, contributed to the variability between studies. This has been previously highlighted as an issue within cancer research (46) as demonstrated in previous reviews examining the impact of morbidity and multimorbidity on breast cancer–screening attendance (8, 10, 12). The high heterogeneity levels could also be related to other factors such as participant ethnicity in included studies, which were largely underreported, yet important to consider for breast cancer and comorbidity outcomes (47). Given that women in ethnic minority groups with breast cancer had higher CCI scores and may be more likely to have multiple conditions, future research should include the ethnicity of participant samples in relation morbidity profiles.

Strengths of this review are that it extends knowledge by examining the impact of individual morbidities and multimorbidity on organized breast cancer–screening attendance and it is the first to quantify the association between different types of morbidity, including disability and respiratory conditions, on breast-screening attendance. Articles included were of high quality ensuring lower bias. Moreover, the study exclusion criteria of self-reported screening, led to all included studies having increased quality scores, although using the NOS aided stratified analysis to exclude those determined to have lower quality. Future reviews could consider excluding studies based on adequacy of morbidity detail. Data only from organized screening programs were used to ensure the influence of increased healthcare contact that may be minimized in comparison with opportunistic programs. Sub-group analyses with morbidity clusters, based on a well-known comorbidity measure used in oncology, allowed for comprehensive reporting of the relative influence of specific groups of conditions. Although results of this review focus only on breast-screening attendance at organized programs, other reviews have illustrated that psychiatric conditions are associated with reduced uptake across geographic regions that include opportunistic screening, such as the US (12). The odds of non-attendance may be even higher for other morbidities due to the systems-level barriers such as health insurance coverage but it is currently unclear.

Limitations include the use of heterogeneous control groups in studies included in this review. For example, some studies had control groups of “healthy” participants (with zero conditions), whereas other control groups did not have the condition in question but individuals had the potential to have other conditions, meaning the impact of other morbidities may have affected screening attendance. Although articles that measured morbidity after screening were excluded, temporality of screening measurement was not always transparently presented. In addition, due to poor reporting across studies, it was not possible to assess whether length of time since diagnosis of morbidity could influence breast-screening attendance, and thus may contribute to the heterogeneity of findings. The high levels of heterogeneity present in the findings, despite conducting sensitivity analyses where possible, limit the strength of the conclusions from this review.

There are several implications from these findings. Despite the heterogeneity of study findings, there is a clear need to assess the subsequent impact of increasing attendance in groups with potentially life shortening morbidity and reduced attendance odds on breast cancer outcomes. Similarly, it is necessary to understand whether decisions not to attend are informed given that breast screening is non-mandatory. For example, breast-screening invitation processes, usually by standardized letter, may not be appropriate for individuals with conditions identified to have the lowest odds of attendance such as neurological conditions, or intellectual disability. Increasing availability of easy-read invitations and literature, at least in some programs, rely on the individual to request these after their initial invite despite linkage with primary care to identify the eligible population. Improved interaction between primary care and screening sites may be tools to improve equity of access at a systems-level to allow individual level decision-making based on appropriate screening information. Similarly, the responsibility of notifying screening programs that more time may be required for an appointment or where there are mobility issues, also typically lies with individuals invited. The reduction of these practical barriers will likely increase equity of access. It may also be appropriate for breast-screening programs to be notified of decisions not to attend screening due to limited overall life expectancy, discussions that may be better facilitated by primary or secondary care providers rather than being the responsibility of the individual alone.

Future research should further explore barriers and facilitators to breast cancer–screening attendance among those with the lowest odds of attendance (individuals with neurological conditions; disability; and mental health conditions). This should also include studies assessing informed choice as it is not currently clear whether this influences breast-screening attendance in these groups (48), particularly in cases where decisions are made by the individuals themselves or by proxy via caregivers. Similarly, interventions that have been developed to improve access to breast screening in general populations, for example, using social media engagement (49), may not be appropriate or even reach some of the morbidity cluster groups that have the greatest odds of non-attendance. Finally, because of the mixed reporting in the studies included in this review of potential sociodemographic confounders, such as ethnicity and deprivation (50), it is unclear whether interventions developed specifically to reduce inequity of access for these reasons would be effective if individuals also have morbidities. The identification of such research gaps can subsequently form the development and testing of theory-based interventions to increase equity of access to breast screening. Furthermore, this may lead to establishing whether increasing attendance would decrease breast cancer–related mortality.

Much heterogeneity of findings was noted in the present research and as previously mentioned, is likely underpinned by the high levels of variability in how morbidity is defined, graded, and identified. For example, defining morbidity based on medication prescription records may not accurately reflect a confirmed diagnosis nor is it information readily available to breast-screening programs despite their responsibility of inviting those eligible to attend. Similarly, severity of conditions such as depression or obesity status could be expected to vary over time for any one individual whereas broad terms such as “brain injury” shed little light on what this means functionally. In addition, “valid” measures of morbidity often used in research, such as the CCI, do not adequately reflect the severity of conditions, particularly those with significant improvements in prevention and management (such as HIV) since the measure was developed. Such measures thereby fail to produce clear assessments of multimorbidity. Given the importance of core outcome sets in research, international consensus is necessary and urgently required to determine optimum sources of morbidity data, linkage between different medical record systems, and definitions to improve transparency and comparison of evidence. This could contribute to the reduction of heterogeneity between future research studies and help explore the interactions between different combinations of co-existing conditions. Of note, only one study included in this analysis considered physical and mental health conditions alongside disability (32). Our literature search identified no studies that considered the impact of comorbidity and reduced attendance at breast screening on subsequent diagnosis. This may be due to the difficulties in linking medical records in databases held in different healthcare systems. Therefore, research is required to determine whether reduced likelihood of screening attendance in individuals with specific comorbidities impacts on breast cancer diagnosis and outcomes.

In conclusion, having any morbidity reduces the odds of breast cancer–screening attendance and is particularly pronounced for individuals with neurological, psychiatric, and disability conditions. Further research should explore reasons for reduced attendance among individuals within these groups to determine whether specific interventions are required with a focus on assessing informed decision making rather than screening attendance alone.

## Supplementary Material

Supplementary Data
