# Supplementary material for: The Impact of Morbidity and Disability on Attendance at Organized Breast Cancer–Screening Programs: A Systematic Review and Meta-Analysis
Source: Cancer Epidemiol Biomarkers Prev. 2022 May 5;31(7):1275–83. doi: 10.1158/1055-9965.EPI-21-1386 (PMC9377755; doi:10.1158/1055-9965.EPI-21-1386)
Supplement: Supplementary Data [file epi-21-1386_supplementary_tables_1-3_and_supplementary_materials_and_methods_suppst.docx]

**The impact of morbidity and disability on attendance at organised breast cancer screening programmes: A systematic review and meta-analysis**

**Supplementary material content**

Supplementary Table 1: Quality Assessment of Included Studies

Supplementary Table 2: Characteristics of Included Studies

Supplementary Table 3: Reasons for Exclusion of Ineligible Studies

Breast Cancer Screening Search Terms

References of Included and Excluded Studies

| **Supplementary Table 1: Quality Assessment of Included Studies** | | | | | | | | | | |
| --- | --- | --- | --- | --- | --- | --- | --- | --- | --- | --- |
| **Study** | **Selection** | | | | **Comparability** | | **Outcome** | | | **Total** |
|  | Representativeness of the exposed cohort | Selection of the non-exposed cohort | Ascertainment of exposure | Temporality Established | Controls for Age | Controls for Additional factors | Assessment of outcome | Was follow-up long enough for outcomes to occur | Adequacy of follow up of cohorts |  |
| Chan 2014 | * | * | * | * | * | * | * | * | * | 9 |
| Chochinov 2009 | * | * | * | * | * | * | * | * | - | 8 |
| Cobigo 2013 | * | * | * | * | * | * | * | * | * | 9 |
| Czwikla 2019 | - | - | * | - | * | * | * | * | - | 5 |
| Floud 2017 | - | * | - | * | * | * | * | * | * | 7 |
| Grunfeld 2012 | * | * | * | * | - | - | * | * | - | 6 |
| Hellmann ‎2015 | - | * | * | * | * | * | * | * | - | 7 |
| Jensen 2015, 2016, Virgilsen 2018 | * | * | * | * | * | * | * | * | * | 9 |
| Katz 2018 | * | * | * | - | * | * | * | * | - | 7 |
| Kendall 2017 | * | * | * | * | * | * | * | * | * | 9 |
| Khan 2010 | * | * | * | * | * | * | * | * | - | 8 |
| Lagerlund 2015 | - | * | * | * | * | * | * | * | * | 8 |
| MB Larsen 2020 | * | * | * | * | * | - | * | * | * | 8 |
| M Larsen 2020 | * | * | - | - | * | * | * | * | - | 5 |
| McCowan ‎2019 | * | * | * | - | * | * | * | * | * | 8 |
| Osborn 2012 | * | * | * | * | * | * | * | * | - | 8 |
| Ouellette-Kuntz ‎2015 | * | * | * | * | - | - | * | * | * | 7 |
| Ross 2019 | * | * | - | * | * | * | * | * | - | 7 |
| Shin 2020b | * | * | * | - | * | * | * | * | * | 8 |
| Singh 2011 | * | * | * | * | * | * | * | * | - | 8 |
| Vigod 2011 | - | * | - | * | * | * | * | * | * | 7 |
| Viuff 2020 | * | * | * | * | * | * | * | * | * | 9 |
| Werneke 2006 | * | * | * | - | - | - | * | * | * | 6 |
| Widdifield 2017 | * | * | * | - | - | - | * | * | * | 6 |
| Woodhead 2016 | - | * | * | - | * | * | * | * | - | 6 |

| **Supplementary Table 2: Characteristics of Included Studies** | | | | | | | | | | | | | | |
| --- | --- | --- | --- | --- | --- | --- | --- | --- | --- | --- | --- | --- | --- | --- |
| **Author** | **Country/ Region** | **Screening time period (years)** | **Study type** | **Location of morbidity data** | **Morbidity cluster** | **Specific morbidity; duration (time); severity** | **N with morbidity** | **N Controls** | **N Total (Extracted for primary outcome)** | **Age Range** | **Adjusted Odds Ratios Y/N** | **Adjusted variables** | **Multimorbidity measurement Y/N** | **Impact on Stage Measured Y/N** |
| Chan 2014^1^ | Ontario Canada | 1999-2010 (One opportunity) | Cohort | Ontario Diabetes Database | Renal/ Endocrine | Diabetes; at least 2 years; not measured | 188,759 | 315,529 | 504,288 | 50-69 | Y | Age, socio-economic status quintile, rural residence, recent immigration into Ontario, comorbidity | N | N |
| Chochinov 2009^2^ | Manitoba Canada | 2002-2004 (One opportunity) | Data linkage | Medical records and claims data | Psychiatric | Schizophrenia ; not specified; classified as ‘severe mental illness’ | 1,448 | 108,792 | 110,240 | 50-69 | Y | Region, age, average household income, continuity of care, physical comorbidities | N | N |
| Cobigo2013^3^ | Ontario Canada | 2008-2010 (One opportunity) | Cohort | Medical records and claims data | Disability | Intellectual and developmental disabilities; not specified; based on ‘Resource Utilization Bands’, expected use of healthcare resources | 5,171 | 488,545 | 493,716 | 50-69 | Y | Age, rurality, income quintile, Resource Utilization Bands | N | N |
| Czwikla 2019^4^ | Lower Saxony Germany | 2011-2014 (Multiple screening opportunities) | Cohort | Medical Records | Cardiovascular  Neurological  Respiratory  Renal/ Endocrine  Gastrointestinal  Rheumatologic/Immunologic  Malignancy  Psychiatric | Congestive heart failure; Cardiac arrhythmias; Valvular disease; Pulmonary circulation disorders; Peripheral vascular disorders; Hypertension; Obesity; not specified; not measured  Paralysis; Other neurological disorders  Chronic Pulmonary disease  Diabetes;Hypothyroidism; Renal failure disease; Fluid and electrolyte disorders  Liver & peptic ulcer disease  AIDS/HIV, Rheumatoid arthritis/collagen vascular diseases  Lymphoma; Metastatic cancer; Solid tumor without metastasis  Alcohol abuse; Drug abuse; Psychoses; Depression | Not stated | Not stated | 96,273 | 50-66 | Y (unable to use in analyses) | Individual and contextual predictors | N | N |
| **Author** | **Country/ Region** | **Screening time period (years)** | **Study type** | **Location of morbidity data** | **Morbidity cluster** | **Specific morbidity duration (time); severity** | **N with morbidity** | **N Controls** | **N Total (Extracted for primary outcome)** | **Age Range** | **Adjusted Odds Ratios Y/N** | **Adjusted variables** | **Multimorbidity measurement Y/N** | **Impact on Stage Measured Y/N** |
| Floud 2017^5^ | England United Kingdom | 2006-2011 (One opportunity) | Cohort (Million Women study) | Self-reported | Disability  Neurological | Hearing Disability; not specified; not measured  Mobility Disability  Vision Disability  Self-care Disability  Memory Disability | 14,555  79,121  6,493  24,253  10,535 | 343,952 | 442,244 | 50-70 | Y (unable to use in analyses) | Age, region, deprivation, ethnicity, marital status, car availability, body mass index, and smoking | Y | N |
| Grunfeld 2012^6^ | Ontario Canada | 1992-2000 (One opportunity) | Cohort | Ontario Cancer Registry | Malignancy | Previous Colorectal Cancer; between 1-5 years from date of diagnosis; not measured  Previous Endometrial Cancer  Previous Hodgkin Lymphoma | 1,489  2,713  151 | 7,441  13,993  762 | 26,549 | 50-79 | N | Not applicable | N | N |
| Hellmann ‎2015^7^ | Copenhagen Denmark | 1993-2008 (One opportunity) | Cohort (Danish Diet, Cancer, and Health cohort) | Measured by researchers | Cardiovascular | Obesity; up to two years; by BMI category | 907 | 2,381 | 3,288 | 50-69 | Y (unable to use in analyses) | Age, birth cohort, education, menarche age, menopausal status & age, hormone replacement therapy use, oral contraceptives use, parity, age first childbirth, breastfeeding, smoking, alcohol, sports, comorbidity | N | N |
| **Author** | **Country/ Region** | **Screening time period (years)** | **Study type** | **Location of morbidity data** | **Morbidity cluster** | **Specific morbidity; duration (time); severity** | **N with morbidity** | **N Controls** | **N Total (Extracted for primary outcome)** | **Age Range** | **Adjusted Odds Ratios Y/N** | **Adjusted variables** | **Multimorbidity measurement Y/N** | **Impact on Stage Measured Y/N** |
| Jensen 2015^8^  Jensen 2016^9^  Virgilsen 2018^10^ | Central Denmark Region Denmark | 2008-2009 (One opportunity) | Cohort | National Patient Registry  National Psychiatric  Central Research Register  National Cancer Registry | Cardiovascular  Renal and endocrine  Respiratory  Neurological  Rheumatologic/Immunologic  Gastrointestinal  Psychiatric  Malignancy | Cardiovascular diseases; not specified; severe multimorbidity specified as having 3+ diseases  Hypertension  Diabetes  Chronic kidney disease  COPD  Chronic neurological disorders  Chronic arthritis  Chronic bowel disease  Chronic liver disease  Schizophrenia; no minimum; not measured  Affective disorders  Anxiety  Substance Abuse  Previous Cancer; no minimum; ‘previous cancer, in treatment, in follow-up, in palliative care’ | 6,463  3,870  2,854  254  2,116  1,137  1,213  1,087  354  1,022  3,453  670  1,427  6,638 | 111,706 | 144,264 | 50-69 | Y (unable to use in analyses) | Age, ethnicity, marital status, education  Age, ethnicity, marital status, education, co-occurrence of other psychiatric diagnoses | Y | N |
| Katz 2018^11^ | Israel | 2008-2014 (Multiple screening opportunities) | Cohort | Medical records | Cardiovascular  Renal/ Endocrine  Psychiatric | Obesity; not specified; not measured  Ischemic heart disease  Diabetes  Depression | 3,614  3,608  5,301  2,510 | 10,193 | 25,226 | 56-74 | Y (unable to use in analyses) | Age, SES, smoking, clinic visits, other comorbidities, physician factors | N | N |
| Kendall 2017^12^ | Ontario Canada | 2011-2013 (One opportunity) | Cross-sectional | Ontario Registered Persons Database | Rheumatologic/Immunologic | HIV; not specified; not measured | 623 | 1,446,392 | 1,447,015 | 50-74 | Y (unable to use in analyses) | Age, income quintile, rurality, comorbidity, immigrant status, physician factors, primary care model | Y | N |
| Khan 2010^13^ | United Kingdom | 2003-2006 (One opportunity) | Cohort | Medical records | Malignancy | Previous Breast cancer; at least 5-year survival; CCI adjusted  Previous Colorectal cancer | 2,317  513 | 9,974  2,055 | 14,859 | 50-69 | Y (unable to use in analyses) | Charlson comorbidity score, history of mastectomy, death | N | N |
| **Author** | **Country/ Region** | **Screening time period (years)** | **Study type** | **Location of morbidity data** | **Morbidity cluster** | **Specific morbidity; duration; severity** | **N with morbidity** | **N Controls** | **N Total (Extracted for primary outcome)** | **Age Range** | **Adjusted Odds Ratios Y/N** | **Adjusted variables** | **Multimorbidity measurement Y/N** | **Impact on Stage Measured Y/N** |
| Lagerlund 2015^14^ | Malmö Sweden | 1992-2009  (Multiple screening opportunities) | Cohort (Malmö Diet and Cancer Study) | Measured by researchers | Cardiovascular | Obesity; range from 0 to 17 years; BMI category | 1,534 | 5,979 | 7,513 | 44-70 | Y | Age, baseline year, season, number of screening invitations after baseline, having been invited to screening before baseline, education, employment status, cohabitation, and country of birth, health-related variables | N | N |
| Larsen 2018^15^ | Central Denmark Region | 2008-2009 (One opportunity) | Cohort | National Administrative Database | Composite morbidity score | CCI; not specified; score of 0, 1 & 2 | 13,653 (not included in total) | 62,867 (not included in total) | Participant data not extracted for analysis | 50-64 | Y (unable to use in analyses) | Age, ethnicity, marital status, household income, education level, CCI score | N | N |
| MB Larsen 2020^16^ | Denmark | 2015-2016 (One opportunity) | Cohort | Danish National Cancer Register | Malignancy | Previous breast cancer; ‘no breast imaging within the past 21 months’; not measured | 17,822 | 662,168 | 679,990 | 50-69 | N | Not applicable | N | N |
| M Larsen 2020^17^ | Norway | 1996-2019 (Multiple screening opportunities) | Cohort | Self-reported | Cardiovascular | Obesity; not specified; BMI category | 5,662 | 16,613 | 22,275 | 50-69 | Y | Education, physical activity, smoking status | N | N |
| McCowan ‎2019^18^ | Scotland United Kingdom | 2009-2013 (One opportunity) | Cohort | Medical records | Disability  Malignancy  Psychiatric | Learning Disability; not specified; not measured  Malignancy  Non-malignant neoplasm  Depression | 629  12,827  11,646  33,427 | 101,464 | 159,993 | 40+ | Y (unable to use in analyses) | Age, index of multiple deprivation quintile, care home residency, other comorbidities | N | N |
| Osborn 2012^19^ | United Kingdom | 1999-2009 (Multiple screening opportunities) | Cohort | Medical records | Disability | Learning Disability; not specified; not measured | 2,956 | 17,354 | 20,310 | 50-64 | Y (unable to use in analyses) | Age, time period, country | N | N |
| **Author** | **Country/ Region** | **Screening time period (years)** | **Study type** | **Location of morbidity data** | **Morbidity cluster** | **Specific morbidity; duration; severity** | **N with morbidity** | **N Controls** | **N Total (Extracted for primary outcome)** | **Age Range** | **Adjusted Odds Ratios Y/N** | **Adjusted variables** | **Multimorbidity measurement Y/N** | **Impact on Stage Measured Y/N** |
| Ouellette-Kuntz ‎2015^20^ | Ontario Canada | 2009-2012 (One opportunity) | Cohort | Disability support programme database | Disability | Intellectual and developmental disabilities; not specified; not measured | 7022 | 402,589 | 409,611 | 50-64 | N | Not applicable | N | N |
| Ross 2019^21^ | Northern Ireland United Kingdom | 2011-2014  (One opportunity) | Cohort (Northern Ireland Longitudinal Study) | Self-reported | Disability  Neurological  Respiratory | Communication; self-reported ‘have lasted or are expected last at least 12 months’; not measured  Blindness /partial sight loss  Mobility /dexterity  Deafness /partial hearing  Learning/ intellectual/ social/ behavioural  Frequent confusion or memory loss  Shortness of breath or difficulty breathing | 551  920  11,695  2,975  483  1,245  6,999 | 36,787 | 61,655 (total n in sample is 57,328 so acknowledge some have multiple morbidities) | 50-70 | Y (unable to use in analyses) | Age, marital status, NSSEC, housing tenure, number of cars, educational attainment, area of residence, presence of other disabilities | Y | N |
| Shin 2020^22^ | South Korea | 2014-2015 (One opportunity) | Data linkage | National disability register | Disability  Neurological  Psychiatric  Renal/ Endocrine  Cardiovascular  Respiratory  Gastrointestinal | Physical disability; unknown; disability by ‘grade’  Visual disability  Hearing disability  Speech/language disability  Intellectual disability  Disability - facial disfigurement  Autism  Disability - brain injury  Epilepsy  Disability - mental disorder  Disability - renal failure  Disability - heart problems  Disability - respiratory problems  Disability - liver disease  Disability – ostomies | 472,192  83,317  92,573  3,619  27,396  830  37  77,882  2,490  39,193  23,015  2,198  2,582  1,971  4,394 | 11,522,435 | 12,356,124 | 40+ | Y | Age, sex, income level, place of residence, calendar year | N | N |
| **Author** | **Country/ Region** | **Screening time period (years)** | **Study type** | **Location of morbidity data** | **Morbidity cluster** | **Specific morbidity; duration; severity** | **N with morbidity** | **N Controls** | **N Total (Extracted for primary outcome)** | **Age Range** | **Adjusted Odds Ratios Y/N** | **Adjusted variables** | **Multimorbidity measurement Y/N** | **Impact on Stage Measured Y/N** |
| Singh 2011^23^ | Manitoba Canada | 2002-2008 (Multiple screening opportunities) | Cohort | University of Manitoba IBD Epid. Database | Gastrointestinal | Inflammatory Bowel Disease; defined as 5 physician contacts and/or hospitalisation, not time; presented as healthcare utilisation in 4 quartiles | 860 | 6,563 | 7,423 | 50-69 | Y | Socioeconomic status and prior unilateral mammogram utilization | N | N |
| Vigod 2011^24^ | Ontario Canada | 2002-2004 (One opportunity) | Cohort (Canadian Community Health Survey) | Self-reported | Psychiatric | Depression; at time of data collection only; not measured | 67 | 1,331 | 1,398 | 50-70 | Y | Age, income level, level of education, geographical variability (urban/rural), medical/ psychiatric comorbidity | N | N |
| Viuff 2020^25^ | Denmark | 2007-2010 (One opportunity) | Cross-sectional | Danish National Patient Registry | Cardiovascular  Neurological  Respiratory  Rheumatologic/ Immunologic  Gastrointestinal  Renal/ Endocrine  Malignancy | Myocardial infarction  Congestive heart failure  Peripheral vascular disease  Cerebrovascular disease  Dementia  Hemiplegia  Chronic pulmonary disease  Connective tissue disease  AIDS  Ulcer disease  Mild liver disease  Moderate to severe liver disease  Diabetes 1 and 2  Diabetes with end organ  Renal disease  Any Tumour  Leukaemia  Lymphoma  Metastatic cancer; stratified data by year of diagnosis; presented data in order of placement in the CCI i.e. greater severity if greater score | 5,468  4,498  8,710  18,038  1,019  716  26,588  13,912  145  7,104  4,801  974  18,403  7,611  3,569  18,822  663  1,697  1,927 | 541,538 | 650,003 (using CCI scores to account for individuals having multiple conditions) | 50-69 | Y (unable to use in analyses) | Age, region of residence, other CCI diseases | N | N |
| **Author** | **Country/ Region** | **Screening time period (years)** | **Study type** | **Location of morbidity data** | **Morbidity cluster** | **Specific morbidity; duration; severity** | **N with morbidity** | **N Controls** | **N Total (Extracted for primary outcome)** | **Age Range** | **Adjusted Odds Ratios Y/N** | **Adjusted variables** | **Multimorbidity measurement Y/N** | **Impact on Stage Measured Y/N** |
| Werneke 2006^26^ | England United Kingdom | 1996–1998 (One opportunity) | Data linkage | Medical records | Psychiatric | Psychosis; not specified; enhanced care described as proxy measure of severity | 49 | 44,195 | 44,244 | 50-64 | N | Not applicable | N | N |
| Widdifield 2017^27^ | Ontario Canada | 1990–2013 (One opportunity) | Cohort | Medical records | Rheumatologic/Immunologic | Rheumatoid Arthritis; not specified; not measured | 451 | 4,440 | 4,891 | 50+ | N | Not applicable | N | N |
|  |  |  |  |  |  |  |  |  |  |  |  |  |  |  |
| Woodhead 2016^28^ | England United Kingdom | 2013(One opportunity) | Data linkage | Medical records | Psychiatric | Serious mental illness (Schizophrenia, Bipolar, Other non-organic Psychoses); not specified; severity defined as inpatient stay, being sectioned, records of difficulty managing physical health and record of emergency healthcare access episodes | 625 | 25,385 | 26,010 | 50-70 | Y | Age, ethnicity, borough-level deprivation, mean annual number of primary consultations | N | N |
|  |  |  |  |  |  | **Totals** | 16,250,556 | 1,408,246 | 17,755,075 |  |  |  |  |  |
| CCI, Charlson Comorbidity Index Score; BMI, Body Mass Index | | | | | | | | | | | | | | |

| **Supplementary Table 3: Reasons for Exclusion of Ineligible Studies** | |
| --- | --- |
| **Study** | **Reason** |
| Burton 1998^29^ | Comorbidity measured after screening opportunity |
| Spithoff 2019^30^ | Comorbidity measured after screening opportunity |
| Aro 1999^31^ | Data not Extractable/ Available |
| Banks 2002^32^ | Data not Extractable/ Available |
| Franck 2020^33^ | Data not Extractable/ Available |
| Aarts 2011^34^ | Impact of comorbidity on attendance not measured |
| Aro 2001^35^ | Impact of comorbidity on attendance not measured |
| Bansal 2012^36^ | Impact of comorbidity on attendance not measured |
| Borrell 1999^37^ | Impact of comorbidity on attendance not measured |
| Dresner 2010^38^ | Impact of comorbidity on attendance not measured |
| Eisinger ‎2015^39^ | Impact of comorbidity on attendance not measured |
| Hafslund 2012^40^ | Impact of comorbidity on attendance not measured |
| Kisley 2013^41^ | Impact of comorbidity on attendance not measured |
| Murto 2018^42^ | Impact of comorbidity on attendance not measured |
| Sutradhar ‎2016^43^ | Impact of comorbidity on attendance not measured |
| Acuna 2017^44^ | Missing/inappropriate control |
| Biswas 2005^45^ | Missing/inappropriate control |
| Castledine 2012^46^ | Missing/inappropriate control |
| Cheung 2018^47^ | Missing/inappropriate control |
| Corkum 2014^48^ | Missing/inappropriate control |
| Goutard 2009^49^ | Missing/inappropriate control |
| Inagaki 2018^50^ | Missing/inappropriate control |
| Kellen 2020^51^ | Missing/inappropriate control |
| Kung 2012^52^ | Missing/inappropriate control |
| Lai 2014^53^ | Missing/inappropriate control |
| Lofters 2019^54^ | Missing/inappropriate control |
| MacLurg 2004^55^ | Missing/inappropriate control |
| Pinto 2010^56^ | Missing/inappropriate control |
| Plourde 2018^57^ | Missing/inappropriate control |
| Sullivan 2003^58^ | Missing/inappropriate control |
| Tesch 2019^59^ | Missing/inappropriate control |
| Wong 2017^60^ | Missing/inappropriate control |
| Yen 2015^61^ | Missing/inappropriate control |
| Duffin 2009^62^ | Not a research publication |
| Glover 2014^63^ | Not a research publication |
| Joel 2016^64^ | Not a research publication |
| Brody 2000^65^ | Not organised programme |
| Castellano ‎2001^66^ | Not organised programme |
| Enright 2018^67^ | Not organised programme |
| Giuliani 2016^68^ | Not organised programme |
| Heyding ‎2005^69^ | Not organised programme |
| Lipscombe 2005^70^ | Not organised programme |
| Lu 2011^71^ | Not organised programme |
| Meguerditchian 2012^72^ | Not organised programme |
| Aguilar Shea 2011^73^ | Self-reported screening |
| Alsuwaida 2013^74^ | Self-reported screening |
| Bao 2018^75^ | Self-reported screening |
| Barbosa 2019^76^ | Self-reported screening |
| Bates 2019^77^ | Self-reported screening |
| Bussiere 2014^78^ | Self-reported screening |
| Choi 2013^79^ | Self-reported screening |
| Fujiwara 2018^80^ | Self-reported screening |
| Lai 2012^81^ | Self-reported screening |
| Maltais 2020^82^ | Self-reported screening |
| Mandelzweig 2017^83^ | Self-reported screening |
| O'Brien ‎2020^84^ | Self-reported screening |
| Park 2012^85^ | Self-reported screening |
| Perea  ‎2012^86^ | Self-reported screening |
| Sakellariou ‎2017^87^ | Self-reported screening |
| Tron 2017^88^ | Self-reported screening |
| Urbanoski ‎2003^89^ | Self-reported screening |
| Wang 2018^90^ | Self-reported screening |
| Guilcher ‎2014^91^ | Specific condition/ composite score not used |
| Jensen 2015^92^ | Specific condition/ composite score not used |
| Majeed 1995^93^ | Specific condition/ composite score not used |
| Mead 2015^94^ | Specific condition/ composite score not used |
| O'Brien 2018^95^ | Specific condition/ composite score not used |
| Rebolj 2020^96^ | Specific condition/ composite score not used |
| Ross 2020^97^ | Specific condition/ composite score not used |
| Yen 2014^98^ | Specific condition/ composite score not used |

| **Reason For Exclusion** | **Study Count** |
| --- | --- |
| Comorbidity measured after screening opportunity | **2** |
| Not a research publication | **2** |
| Data not Extractable/ Available | **3** |
| Specific condition/ composite score not used | **8** |
| Not organised programme | **8** |
| Impact of comorbidity on attendance not measured | **10** |
| Missing/inappropriate control | **18** |
| Self-reported screening | **19** |

**Supplementary File: Breast Cancer Screening Search Terms**

#1

Breast and (cancer OR neoplasm OR carcinoma OR tumor* OR tumour* OR malig*) OR *Breast Neoplasms/ [mesh] OR Mammo*

#2

Screen* adj2 (breast OR cancer) OR Breast AND ultrasonogra* OR 3D mammogr* OR *Mammography [MESH]/ or mammogr* OR "breast tomosynthesis" OR "breast magnetic resonance imaging"

#3

attend* OR non-attend* OR adher* OR non-adher* OR participat* OR non-participat* OR uptake OR utilis* OR utiliz* OR compliance OR comply OR non-complet* OR underutiliz* OR underutilis* OR usage OR nonreceipt OR receipt OR disparit* OR take-up OR access OR (up-to-date) OR (rate* or recei*) adj3 (screening) OR (hav* or recei*) adj2 (mammog*) OR (Screening or mammog*) adj (patterns or practices OR behavio?r*) OR “mammog* use”

#4

(Comorbid* OR co-morbid* OR multimorbid* OR multi-morbid*) OR (concomitant adj2 disease*) OR (chronic* adj2 (disease* OR ill* OR condition*)) comorbidity/ [MESH] OR multimorbidity/ [MESH]

Hypertens* OR angina OR obesity OR BMI OR (arterial OR artery OR cardiovascular OR cerebrovascular OR venous OR heart OR coronary) adj1 disease* OR “Heart attack*” OR "myocardial infarction" OR “atrial fibrillation" OR “body mass index”

Asthma OR COPD OR bronchitis OR (Respiratory OR lung OR pulmonary) adj2 disease* OR “Chronic Obstructive Pulmonary Disease"

Dementia OR Alzheimer* OR epilep* OR stroke OR paralys* OR hemiplegi* OR paraplegi* OR (cogniti* OR Neuromuscular OR neurodegenerative OR neurologic* OR neurocog*) adj2 (disorder* OR disease* OR impair* OR condition*) OR "multiple sclerosis"

HIV OR arthrit* OR osteoarthritis OR "Human Immunodeficiency Virus" OR ("Connective tissue”) adj (disease* or disorder*) OR “osteomuscular disease*” OR “acquired immune deficiency syndrome”

((renal OR kidney) adj2 (fail* OR disease* OR insufficiency OR dysfunction*)) OR (dialysis OR diabet* OR CKD) OR “thyroid disease*”

Dyslipid?emia OR IBD OR hepatitis OR pancreatitis OR ulcer OR (digest* adj2 disease*) OR (liver adj2 disease*) OR (bowel adj2 disease*)

((mental* or psychiatric or mood OR affective) adj (ill* or disorder* or health or condition*))

Depress* OR schiz* OR psychosis OR psychoses OR psychotic) OR ((substance OR drug OR opioid OR alcohol) adj1 (misu* OR abus* OR disorder* OR addict* OR depend*)) OR "substance use disorder*” OR “anxiety disorder*”

“Metastatic cancer” OR “Metastatic solid tumo?r” OR “Hodgkin's disease” OR “previous cancer” OR “cancer survivors” OR “secondary cancer”

Development* disab* OR deaf* OR autis* OR disabilit* OR ((disab* OR impair* OR limitation* OR defect*) adj (learning OR intellect* OR physical OR mobility OR neurodevelopmental OR hearing OR visual OR vision OR sensory OR functional)) OR (spinal adj2 injur*) OR (Brain adjInjur*) OR "cerebral palsy"

#1 AND #2 AND #3 AND #4AND Publication date from 01.01.1998-16.09.20

**Supplementary File: References of included and excluded studies**

1. Chan W, Yun L, Austin P, Jaakkimainen R, Booth G, Hux J, et al. Impact of socio‐economic status on breast cancer screening in women with diabetes: a population‐based study. *Diabet Med* 2014; **31**(7): 806-12.

2. Chochinov HM, Martens PJ, Prior HJ, Fransoo R, Burland E. Does a diagnosis of schizophrenia reduce rates of mammography screening? A Manitoba population-based study. *Schizophren Res* 2009; **113**(1): 95-100.

3. Cobigo V, Ouellette‐Kuntz H, Balogh R, Leung F, Lin E, Lunsky Y. Are cervical and breast cancer screening programmes equitable? The case of women with intellectual and developmental disabilities. *J Intellect Disabil Res* 2013; **57**(5): 478-88.

4. Czwikla J, Urbschat I, Kieschke J, Schüssler F, Langner I, Hoffmann F. Assessing and explaining geographic variations in mammography screening participation and breast cancer incidence. *Frontiers Oncol* 2019;9:909.

5. Floud S, Barnes I, Verfürden M, Kuper H, Gathani T, Blanks R, et al. Disability and participation in breast and bowel cancer screening in England: A large prospective study. *Br J Cancer* 2017; **117**(11): 1711-4.

6. Grunfeld E, Moineddin R, Gunraj N, Del Giudice ME, Hodgson DC, Kwon JS, et al. Cancer screening practices of cancer survivors: population-based, longitudinal study. *Can Fam Physician* 2012; **58**(9): 980-6.

7. Hellmann SS, Njor SH, Lynge E, von Euler-Chelpin M, Olsen A, Tjønneland A, et al. Body Mass Index and participation in organized mammographic screening: A prospective cohort study. *BMC Cancer* 2015; **15**(1): 1-9.

8. Jensen LF, Pedersen AF, Andersen B, Vestergaard M, Vedsted P. Non-participation in breast cancer screening for women with chronic diseases and multimorbidity: a population-based cohort study. *BMC Cancer* 2015; **15**(1): 1-10.

9. Jensen LF, Pedersen AF, Bech BH, Andersen B, Vedsted P. Psychiatric morbidity and non-participation in breast cancer screening. *The Breast* 2016; **25**: 38-44.

10. Virgilsen LF, Pedersen AF, Andersen B, Vedsted P. Non-participation in breast cancer screening among previous cancer patients. *J Cancer Res Clin Oncol* 2018; **144**(10): 1959-66.

11. Katz D, Tengekyon AJ, Kahan NR, Calderon-Margalit R. Patient and physician characteristics affect adherence to screening mammography: A population-based cohort study. *PloS one* 2018; **13**(3): e0194409.

12. Kendall CE, Walmsley S, Lau C, Jembere N, Burchell AN, Loutfy M, et al. A cross-sectional population-based study of breast cancer screening among women with HIV in Ontario, Canada. *CMAJ Open* 2017;**5**(3):E673.

13. Khan N, Carpenter L, Watson E, Rose P. Cancer screening and preventative care among long-term cancer survivors in the United Kingdom. *Br J Cancer* 2010; 102(7): 1085-90.

14. Lagerlund M, Drake I, Wirfält E, Sontrop JM, Zackrisson S. Health-related lifestyle factors and mammography screening attendance in a Swedish cohort study. *European Journal of Cancer Prevention* 2015; **24**(1): 44-50.

15. Larsen SH, Virgilsen LF, Kristiansen BJ, Andersen B, Vedsted P. Strong association between cervical and breast cancer screening behaviour among Danish women: A register-based cohort study. *Prev Med Reports* 2018; **12**: 349-354.

16. Larsen MB, Vejborg I, Njor SH. Participation in breast cancer screening among breast cancer survivors–A nationwide register-based cohort study. *The Breast* 2020; **54**: 31-6.

17. Larsen M, Moshina N, Sagstad S, Hofvind S. Factors associated with attendance and attendance patterns in a population-based mammographic screening program. *Journal of Medical Screening* 2020: 0969141320932945.

18. McCowan C, McSkimming P, Papworth R, Kotzur M, McConnachie A, Macdonald S, et al. Comparing uptake across breast, cervical and bowel screening at an individual level: a retrospective cohort study. *Br J Cancer* 2019; **121**(8):710-4.

19. Osborn DP, Horsfall L, Hassiotis A, Petersen I, Walters K, Nazareth I. Access to cancer screening in people with learning disabilities in the UK: Cohort study in the health improvement network, a primary care research database. *PloS one* 2012; **7**(8): e43841.

20. Ouellette‐Kuntz H, Cobigo V, Balogh R, Wilton A, Lunsky Y. The uptake of secondary prevention by adults with intellectual and developmental disabilities. *J Appl Res Intellect Disabil* 2015; **28(**1): 43-54.

21. Ross E, Maguire A, Donnelly M, Mairs A, Hall C, O’Reilly D. Disability as a predictor of breast cancer screening uptake: A population-based study of 57,328 women. *J Med Screen* 2020; **27**(4): 194-200.

22. Shin DW, Yu J, Cho J, Lee SK, Jung JH, Han K, et al. Breast cancer screening disparities between women with and without disabilities: A national database study in South Korea. *Cancer* 2020; **126**(7): 1522-9.

23. Singh H, Nugent Z, Demers AA, Bernstein CN. Screening for cervical and breast cancer among women with inflammatory bowel disease: A population‐based study 1. *Inflammatory Bowel Diseases* 2011; **17**(8):1741-50.

24. Vigod SN, Kurdyak PA, Stewart DE, Gnam WH, Goering PN. Depressive symptoms as a determinant of breast and cervical cancer screening in women: a population-based study in Ontario, Canada. *Arch Women's Ment Health* 2011; **14**(2): 159-68.

25. Viuff JH, Vejborg I, Schwartz W, Bak M, Mikkelsen EM. Morbidity as a predictor for participation in the danish national mammography screening program: A cross-sectional study. *Clinical Epidemiology* 2020; **12**: 509.

26. Werneke U, Horn O, Maryon-Davis A, Wessely S, Donnan S, McPherson K. Uptake of screening for breast cancer in patients with mental health problems. *J Epidemiol Community Health* 2006; **60**(7): 600-5.

27. Widdifield J, Ivers NM, Bernatsky S, Jaakkimainen L, Bombardier C, Thorne JC, et al. Primary care screening and comorbidity management in rheumatoid arthritis in Ontario, Canada. *Arthritis Care & Research* 2017; **69**(10): 1495-503.

28. Woodhead C, Cunningham R, Ashworth M, Barley E, Stewart RJ, Henderson MJ. Cervical and breast cancer screening uptake among women with serious mental illness: A data linkage study. *BMC Cancer* 2016; **16**(1): 1-9.

29. Burton MV, Warren R, Price D, Earl H. Psychological predictors of attendance at annual breast screening examinations. *Br J Cancer* 1998; **77**(11): 2014-9.

30. Spithoff S, Kiran T, Khuu W, Kahan M, Guan Q, Tadrous M, et al. Quality of primary care among individuals receiving treatment for opioid use disorder. *Can Fam Physician* 2019; **65**(5): 343-51.

31. Aro AR, de Koning HJ, Absetz P, Schreck M. Psychosocial predictors of first attendance for organised mammography screening. *J Med Screen* 1999; **6**(2): 82-8.

32. Banks E, Beral V, Cameron R, Hogg A, Langley N, Barnes I, et al. Comparison of various characteristics of women who do and do not attend for breast cancer screening. *Breast Cancer Res* 2002; **4**(1): R1.

33. Franck JE, Ringa V, Rigal L, Sassenou J, Coeuret-Pellicer M, Chauvin P, et al. Patterns of gynaecological check-up and their association with Body Mass Index within the CONSTANCES cohort. *J Med Screen* 2020: 969141320914323.

34. Aarts M, Voogd A, Duijm LE, Coebergh J, Louwman W. Socioeconomic inequalities in attending the mass screening for breast cancer in the south of the Netherlands—associations with stage at diagnosis and survival. *Breast Cancer Res Treat* 2011; **128**(2): 517-25.

35. Aro AR, de Koning HJ, Absetz P, Schreck M. Two distinct groups of non-attenders in an organized mammography screening program. *Breast Cancer Res Treat* 2001; **70**(2): 145-53.

36. Bansal N, Bhopal RS, Steiner MF, Brewster DH, Scottish H, Ethnicity Linkage S. Major ethnic group differences in breast cancer screening uptake in Scotland are not extinguished by adjustment for indices of geographical residence, area deprivation, long-term illness and education. *Br J Cancer* 2012; **106**(8): 1361-6.

37. Borrell C, Rohlfs I, Ferrando J, Pasarin MI, Dominguez-Berjon F, Plasencia A. Social inequalities in perceived health and the use of health services in a southern European urban area. *Int J Health Serv* 1999; **29**(4): 743-64.

38. Dresner Y, Frank E, Baevsky T, Rotman E, Vinker S. Screening practices of Israeli doctors' and their patients. *Prev Med* 2010; **50**(5-6): 300-3.

39. Eisinger F, Viguier J, Touboul C, Coscas Y, Pivot X, Blay JY, et al. Social stratification, risk factor prevalence and cancer screening attendance. *Eur J Cancer Prev* 2015; **24** (Suppl:S77-81).

40. Hafslund B, Espehaug B, Nortvedt MW. Health-related quality of life, anxiety and depression related to mammography screening in Norway. *J Clin Nurs* 2012; **21**(21-22): 3223-34.

41. Kisely S, Crowe E, Lawrence D. Cancer-related mortality in people with mental illness. *JAMA Psychiatry* 2013; **70**(2): 209-17.

42. Murto MO, Artama M, Pukkala E, Visvanathan K, Murtola TJ. Breast cancer extent and survival among diabetic women in a Finnish nationwide cohort study. *Int J Cancer* 2018; **142**(11): 2227-33.

43. Sutradhar R, Gu S, Glazier RH, Paszat LF, investigators of the Ontario Cancer Screening Research N. The association between visiting a primary care provider and uptake of periodic mammograms as women get older. *J Med Screen* 2016; **23**(2): 83-8.

44. Acuna SA, Sutradhar R, Camacho X, Daly C, Del Giudice ME, Kim SJ, et al. Uptake of cancer screening tests among recipients of solid organ transplantation. *Am J Transplant* 2017; **17**(9): 2434-43.

45. Biswas M, Whalley H, Foster J, Friedman E, Deacon R. Women with learning disability and uptake of screening: audit of screening uptake before and after one to one counselling. *J Public Health* 2005; **27**(4): 344-7.

46. Castledine CI, Gilg JA, Rogers C, Ben-Shlomo Y, Caskey FJ. How much of the regional variation in RRT incidence rates within the UK is explained by the health needs of the general population? *Nephrol Dial Transplant* 2012; **27**(10): 3943-50.

47. Cheung MC, Tinmouth J, Austin PC, Fischer HD, Fung K, Singh S. Screening for a new primary cancer in patients with existing metastatic cancer: a retrospective cohort study. *CMAJ Open* 2018; **6**(4): E538-E43.

48. Corkum M, Urquhart R, Kephart G, Hayden JA, Porter G. Breast and cervical cancer screening behaviours among colorectal cancer survivors in Nova Scotia. *Curr Oncol* 2014; **21**(5): e670-7.

49. Goutard S, Baron C, Bouton C, Penisson-Besnier I, Fosse G, Aube-Nathier AC, et al. Contraception and screening for cervical and breast cancer in neuromuscular disease: a retrospective study of 50 patients monitored at a clinical reference centre. *Ann Phys Rehabil Med* 2009; **52**(7-8): 538-45.

50. Inagaki M, Fujiwara M, Nakaya N, Fujimori M, Higuchi Y, Hayashibara C, et al. Low cancer screening rates among japanese people with schizophrenia: A cross-sectional study. *Tohoku J Exp Med* 2018; **244**(3): 209-18.

51. Kellen E, Nuyens C, Molleman C, Hoeck S. Uptake of cancer screening among adults with disabilities in Flanders (Belgium). *J Med Screen* 2020; **27**(1): 48-51.

52. Kung PT, Tsai WC, Chiou SJ. The assessment of the likelihood of mammography usage with relevant factors among women with disabilities. *Res Dev Disabil* 2012; **33**(1): 136-43.

53. Lai HT, Kung PT, Tsai WC. Factors influencing the mammography utilization among Taiwanese women with intellectual disabilities, a nationwide population-based study. *Res Dev Disabil* 2014; **35**(12): 3372-8.

54. Lofters A, Chaudhry M, Slater M, Schuler A, Milligan J, Lee J, et al. Preventive care among primary care patients living with spinal cord injury. *J Spinal Cord Med* 2019; **42**(6): 702-8.

55. Maclurg K, Reilly P, Hawkins S. Participation in general practice health screening by people with multiple sclerosis. *Br J Gen Pract* 2004; **54**(508): 853-5.

56. Pinto R, Ashworth M, Seed P, Rowlands G, Schofield P, Jones R. Differences in the primary care management of patients with psychosis from two ethnic groups: A population-based cross-sectional study. *Fam Pract* 2010; **27**(4): 439-46.

57. Plourde N, Brown HK, Vigod S, Cobigo V. The association between continuity of primary care and preventive cancer screening in women with intellectual disability. *Am J Intellect Dev Disabil* 2018;**123**(6) :499-513.

58. Sullivan SG, Glasson EJ, Hussain R, Petterson BA, Slack-Smith LM, Montgomery PD, et al. Breast cancer and the uptake of mammography screening services by women with intellectual disabilities. *Prev Med* 2003; **37**(5): 507-12.

59. Tesch M, Laing K. Screening for new primary cancers in patients with metastatic breast cancer: a provincial analysis of the Choosing Wisely Canada recommendations. *Curr Oncol* 2019; **26**(3): e309-e13.

60. Wong G, Hayward JS, McArthur E, Craig JC, Nash DM, Dixon SN, et al. Patterns and predictors of screening for breast and cervical cancer in women with ckd. *Clin J Am Soc Nephrol* 2017; **12**(1): 95-104.

61. Yen SM, Kung PT, Tsai WC. Mammography usage with relevant factors among women with mental disabilities in Taiwan: A nationwide population-based study. *Res Dev Disabil* 2015; **37**: 182-8.

62. Duffin C. Passing the screen test. *Learning Disability Practice* 2009; **12**(1):11.

63. Glover G. Access to cancer screening by people with learning disabilities in England 2012/13: Information from the Joint Health and Social Care Assessment Framework. *Tizard Learning Disability Review* 2014; **19**(4): 1-13.

64. Joel M. Cancer screening: better access for service users. *Learning Disability Practice* 2016; **19**(5): 16-23.

65. Brody ER, Kohler SA, Rask KJ. Physician and patient characteristics associated with adherence to preventive care guidelines. *Journal of Clinical Outcomes Management* 2000; **7**(3): 25-32.

66. Castellano PZ, Wenger NK, Graves WL. Adherence to screening guidelines for breast and cervical cancer in postmenopausal women with coronary heart disease: an ancillary study of volunteers for hers. *J Womens Health Gend Based Med* 2001; **10**(5): 451-61.

67. Enright K, Desai T, Sutradhar R, Gonzalez A, Powis M, Taback N, et al. Factors associated with imaging in patients with early breast cancer after initial treatment. *Curr Oncol* 2018; **25**(2): 126-32.

68. Giuliani O, Mancini S, Puliti D, Caranci N, Ravaioli A, Vattiato R, et al. Patterns and determinants of receipt of follow-up mammography and/or clinical examination in a cohort of Italian breast cancer survivors. *Breast Cancer Res Treat* 2016; **158**(3): 543-51.

69. Heyding RK, Cheung AM, Mocarski EJ, Moineddin R, Hwang SW. A community-based intervention to increase screening mammography among disadvantaged women at an inner-city drop-in center. *Women Health*. 2005; **41**(1): 21-31.

70. Lipscombe LL, Hux JE, Booth GL. Reduced screening mammography among women with diabetes. *Arch Intern Med* 2005; **165**(18): 2090-5.

71. Lu W, Jansen L, Schaapveld M, Baas PC, Wiggers T, De Bock GH. Underuse of long-term routine hospital follow-up care in patients with a history of breast cancer? *BMC Cancer* 2011; **11**:279.

72. Meguerditchian AN, Dauphinee D, Girard N, Eguale T, Riedel K, Jacques A, et al. Do physician communication skills influence screening mammography utilization? *BMC Health Serv Res* 2012; **12**:219.

73. Aguilar Shea AL, Vera Garcia M, Outomuro Cadavid A, Cabrera Velez R, Martin Diaz M, Bernardo Fernandez T. Health of family physicians: do we practice what we preach?. *Aten Primaria* 2011; **43**(6): 305-11.

74. Alsuwaida A, AlSharidi A, AlAnazi N, AlGhamdi M, AlMeshal M, AlJaser R, et al. Health and well-being among physicians. *Intern Med J* 2013; **43**(12): 1310-5.

75. Bao HL, Wang LH, Wang LM, Fang LW, Zhang M, Zhao ZP, et al. Study on the coverage of cervical and breast cancer screening among women aged 35-69 years and related impact of socioeconomic factors in China, 2013. *Chung Hua Liu Hsing Ping Hsueh Tsa Chih* 2018; **39**(2): 208-12.

76. Barbosa YC, Oliveira AGC, Rabelo PPC, Silva FS, Santos AMD. Factors associated with lack of mammography: National Health Survey, 2013. *Rev Bras Epidemiol* 2019; **22**: e190069.

77. Bates C, Triantafyllopoulou P. Exploring the impact of mental capacity on breast screening for women with intellectual disabilities. *Health Soc Care Community* 2019;**27** (4): 880-8.

78. Bussiere C, Sicsic J, Pelletier-Fleury N. The effects of obesity and mobility disability in access to breast and cervical cancer screening in france: results from the national health and disability survey. *PLoS one*. 2014; **9**(8): e104901.

79. Choi KH, Heo J, Kim S, Jeon YJ, Oh M. Factors associated with breast and cervical cancer screening in Korea: data from a national community health survey. *Asia Pac J Public Health* 2013; **25**(6): 476-86.

80. Fujiwara M, Inagaki M, Nakaya N, Fujimori M, Higuchi Y, Kakeda K, et al. Association between serious psychological distress and nonparticipation in cancer screening and the modifying effect of socioeconomic status: Analysis of anonymized data from a national cross-sectional survey in Japan. *Cancer* 2018; **124**(3): 555-62.

81. Lai CY, Lai CM, Chen CY, Koo M. Factors associated with non-utilization of mammographic screening services in middle-aged and elderly women in Taiwan. *Hu Li Tsa Chih* 2012; **59**(2): 61-71.

82. Maltais J, Morin D, Tasse MJ. Healthcare services utilization among people with intellectual disability and comparison with the general population. *J Appl Res Intellect Disabil* 2020; **33**(3): 552-64.

83. Mandelzweig L, Chetrit A, Amitai T, Silverman B, Siegelmann-Danieli N, Sadetzki S. Primary prevention and screening practices among long-term breast cancer survivors. *Cancer Causes Control* 2017; **28**(7): 657-66.

84. O'Brien N, Godard-Sebillotte C, Skerritt L, Dayle J, Carter A, Law S, et al. Assessing gaps in comprehensive HIV care across settings of care for women living with HIV in Canada. *J Womens Health* 2020; **05**: 05.

85. Park JK, Park HA, Park JJ, Cho YG. Obesity and screening compliance for breast and cervical cancer in Korean women. *Asian Pac J Cancer Prev* 2012; **13**(7): 3271-4.

86. Perea MD, Castano-Vinyals G, Altzibar JM, Ascunce N, Moreno V, Tardon A, et al. Cancer screening practices and associated lifestyles in population controls of the Spanish multi-case control study. *Gac Sanit* 2012; **26**(4): 301-10.

87. Sakellariou D, Rotarou ES. Utilisation of cancer screening services by disabled women in Chile. *PLoS one* 2017; **12**(5): e0176270.

88. Tron L, Lert F, Spire B, Dray-Spira R, Agence Nationale de Recherche sur le Sida et les Hepatites Virales -Vespa2 Study G. Levels and determinants of breast and cervical cancer screening uptake in HIV-infected women compared with the general population in France. *HIV Med* 2017; **18**(3): 181-95.

89. Urbanoski KA, Urbanoski KA. The use of preventive healthcare by Canadian women who drink alcohol. *Prev Med* 2003; **37**(4): 334-41.

90. Wang KH, Thompson TA, Galusha D, Friedman H, Nazario CM, Nunez M, et al. Non-communicable chronic diseases and timely breast cancer screening among women of the Eastern Caribbean Health Outcomes Research Network (ECHORN) Cohort Study. *Cancer Causes Control* 2018; **29**(3): 315-24.

91. Guilcher SJ, Lofters A, Glazier RH, Jaglal SB, Voth J, Bayoumi AM. Level of disability, multi-morbidity and breast cancer screening: does severity matter? *Prev Med* 2014; **67**: 193-8.

92. Jensen LF, Pedersen AF, Andersen B, Vedsted P. Self-assessed health, perceived stress and non-participation in breast cancer screening: A Danish cohort study. *Prev Med* 2015; **81**: 392-8.

93. Majeed FA, Cook DG, Given-Wilson R, Vecchi P, Poloniecki J. Do general practitioners influence the uptake of breast cancer screening? *J Med Screen* 1995; **2**(3): 119-24.

94. Mead L, Porteous L, Tait M, Stoker R, Payne S, Calvert C, et al. The prevalence of medical reasons for non-participation in the Scottish breast and bowel cancer screening programmes. *J Med Screen* 2015; **22**(2): 106-8.

95. O'Brien KM, Mooney T, Fitzpatrick P, Sharp L. Screening status, tumour subtype, and breast cancer survival: A national population-based analysis. *Breast Cancer Res Treat* 2018; **172**(1): 133-42.

96. Rebolj M, Parmar D, Maroni R, Blyuss O, Duffy SW. Concurrent participation in screening for cervical, breast, and bowel cancer in England. *J Med Screen* 2020; **27**(1): 9-17.

97. Ross E, Maguire A, Donnelly M, Mairs A, Hall C, O'Reilly D. Does poor mental health explain socio-demographic gradients in breast cancer screening uptake? A population-based study. *Eur J Public Health* 2020; **30**(3): 396-401.

98. Yen SM, Kung PT, Chiu LT, Tsai WC. Related factors and use of free preventive health services among adults with intellectual disabilities in Taiwan. *BMC Health Serv Res* 2014; **14**(1): 248.
